# Evaluation of synergy between low-carbon development and socio-economic development based on a composite system: a case study of Anhui Province (China)

**DOI:** 10.1038/s41598-022-24937-5

**Published:** 2022-11-24

**Authors:** Ziwei Yang, Jingli Liu, Qinfeng Xing

**Affiliations:** State Key Laboratory of Mining Response and Disaster Prevention and Control in Deep Coal Mines, Anhui University of Science and Technology, Huainan, 232001 Anhui China

**Keywords:** Environmental sciences, Environmental social sciences

## Abstract

Low-carbon development has been the favorite subject with the aim of making a synergy environment. Extensive research on low-carbon development and socio-economic synergy has been conducted by scholars from different perspectives. This paper explores the synergy between low-carbon development and socio-economic development as a composite system from four levels: resource, energy, economy and society, and improves the theory of low-carbon economic development in a "dual-carbon" perspective. Meanwhile, based on the theoretical research, this paper constructs a synergy model and evaluation index system of low-carbon development and socio-economic development, and conducts an empirical analysis by combining the relevant data of low-carbon governance and socio-economic development in Anhui Province from 2011 to 2020. After obtaining the level of synergistic development and constraints of the composite system, the trend of changes in the level of synergy of the composite system is verified by R/S model. It is found that: (1) the composite system composed of low-carbon development and socio-economic development in Anhui Province has certain synergistic effects, and the level of synergistic development is increasing; (2) the studied time series has significant correlation over a long period of time, while it will continue the past development trend. The contribution of this paper is to take the synergy model as the basis and build the R/S model to extend the time cycle of synergy level, which extends the previous theoretical research system of low-carbon development, helps to deepen the theoretical understanding of the synergy between low-carbon development and socio-economic development, and improves the discussion on building ecological civilization and helping to achieve the goal of "dual-carbon".

## Introduction

With the continuous modernization process, the strategic position of ecological civilization construction and ecological environmental protection has become more prominent, and taking the low-carbon sustainable development path has become a consensus. It is undeniable that green low-carbon and socio-economic win–win is the only way to achieve sustainable development^1^. Obviously, economic development cannot be separated from new economic growth points and the composition of industrial structure, but in order to meet the requirements of synergistic win–win economic and environmental development, the innovation of development model and the choice of development path are inseparable. Therefore, in order to achieve the synergistic development of environmental protection and socio-economic development, it is necessary to change the traditional development mode from sustainable development, improve the energy utilization rate, adjust the industrial structure and energy structure, build a green, low-carbon and circular development economic system, and then form a new pattern of modernization and construction for the harmonious development of human and nature^2^. Therefore, based on the above background, this paper constructs a low-carbon development system containing energy system and resource system through a synergy model, which constitutes a composite system with the socio-economic development system and verifies the synergy between the two.

In order to successfully carry out the relevant research and achieve the research objectives, the paper is prepared by combing the previous literature accordingly. First, in the construction of the composite system evaluation index system, it is especially important to select appropriate indicators for the study. The factors affecting the low-carbon economy in China mainly include industrial structure, energy structure and efficiency, openness to the outside world, per capital GDP, residential consumption level, and fiscal revenue, in addition to economic development mode, energy consumption structure, industrialization and urbanization, energy consumption intensity, and lifestyle^3,4^. The factors affecting low-carbon development can be divided into five dimensions: low-carbon economy, low-carbon technology, low-carbon environment, low-carbon ecology, and low-carbon life^5^. Under the goal of "dual-carbon", optimal adjustment of energy structure, industrial structure, production and consumption structure, rational planning of afforestation, and active promotion of international carbon emissions trading have become the core strategies to promote the green and low-carbon development of China's economy. Second, the measurement tools are also key in the study of the level of synergy in the development of the composite system. Panel regression models and mediating effect models can be used to analyze the synergistic effects of pollution reduction and carbon reduction policies^6^. The correlation coefficient matrix method and the composite system synergy model can be used to measure the degree of synergy in the collaborative governance of pollution reduction and carbon reduction in urban agglomerations^7^. The essence of low-carbon development can be clarified in terms of energy flow and resource flow, and then studied by the synergy model and assessment index system. Finally, in terms of models used in trend studies, R/S models are widely used in stock forecasting, runoff forecasting, and other regular data forecasting aspects, and can also be used for forecasting content robustness testing^8–11^. Gray forecasting models are used for carbon emission forecasting, income forecasting, etc.^12^.

Through the above review, it is found that the established literature has studied the synergistic relationship between low-carbon and and socio-economic development from different perspectives, but in terms of indicator selection, few scholars have placed all four—resources, energy, society and economy—within the same assessment system dimension. Theoretical foundation and practice tell us that low-carbon economy can be achieved by optimizing the layout of industrial structure, reducing the efficiency of energy use, and increasing the cultivation of new industries^13–15^. Low-carbon development can be promoted through energy saving and emission reduction, reducing environmental pollution, and increasing carbon sources. By effectively improving the investment environment for talents and developing new industries can effectively improve people's livelihood and realize the synergy between economic and social development and environmental protection. Therefore, the analysis of low carbon development and socio-economic development as a composite system using the synergy model can help to comprehensively understand the connotation and extension of low-carbon economy. The R/S model has been widely used to verify the memory of data cycles, but no research has been applied to the composite system of low-carbon development and socio-economic development.In terms of empirical methods, most of the existing studies have combined industrial structure or energy intensity to analyze low-carbon emission reduction, but few have considered them in the same framework. This paper examines the synergistic effects of low-carbon economy and socio-economic in a more comprehensive way by considering resources, energy, society and economy as four dimensions in the evaluation system. At the same time, the data related to low-carbon and socio-economic development in Anhui Province from 2011 to 2020 are selected to analyze the orderliness of each subsystem and the composite system, calculate the synergy of the composite system, and then use the R/S model to verify the trend of the synergistic effect of the composite system, and use the analysis results to propose the development path and direction to promote the realization of low-carbon economy.

## Materials and methods

### Study area

**Figure 1:**
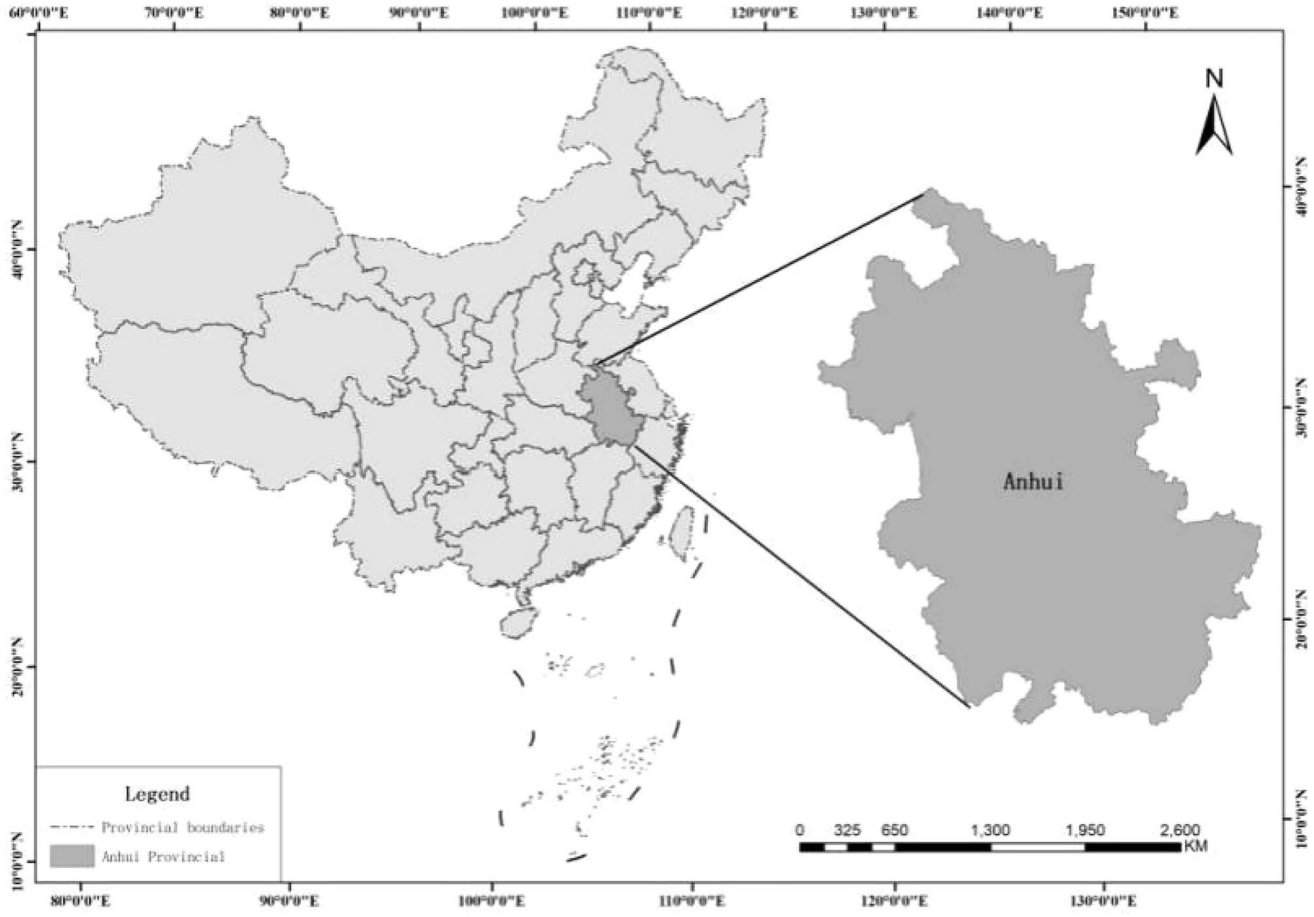
Located of Anhui Province in China.

Anhui Province (shown in Fig. 1) is located in east China, and in the middle and lower reaches of the Yangtze River and Huaihe River, and the hinterland of the Yangtze River Delta. It belongs to the central and eastern economic zones, and the Yangtze River Delta urban agglomeration is formed together with Jiangsu Province, Zhejiang Province and Shanghai City has become one of the six world-class urban agglomerations in the world^16^. The differences among geographical location, human environment and resource endowment are the main reasons for the unbalanced regional development in Anhui Province. In Anhui Province, Wanjiang Economic Zone, South Anhui and north Anhui respectively take high technology industry. Furthermore, tourism and coal mine economy are as the main industries to promote socio-economic development. On the whole, the current situation of low carbon development in Anhui Province is complex and should be further analyzed.

### Data resource

The research data in this study are got from Anhui Provincial Bureau of Statistics, Anhui Provincial Department of Ecology and Environment and Anhui Statistical Yearbook (2011–2020).

### Assessment indicator system construction

#### Indicator selection

Based on the previous low-carbon theory, in order to be able to objectively evaluate the synergistic effectiveness of low-carbon economic development in Anhui Province, with the fundamental principles of scientific and accessibility, the relevant indicators of resource environment and energy environment are adopted as the assessment content of low-carbon development system, and the commonly used macroeconomic measurement indicators are adopted as the assessment content of economic and social development system. Combined with the research contents, the 12 indicators are selected from the four levels of resource environment, energy environment, socio-economic as the evaluation content of subsystem and composite system in Table 1. Thus constituting the low-carbon development indicator system structure of the socio-economic development system (Table 1). Then, this study defines the compound system $S = \left\{ {S_{1} ,S_{2} } \right\}$. Where, $S_{1}$ and $S_{2}$ represent low-carbon development system and socio-economic development system respectively.

**Table 1:** Evaluation indicator of composite system.

–		Target system	Sub-system	Indicator	Variable name
Low-carbon development and socio-economic development system	Low-carbon development system		Resource environment subsystem	Forest coverage	C1
				Air quality indicator good and good rate	C2
				Garbage harmless treatment rate	C3
			Energy environment subsystem	Energy production structure	C4
				Energy consumption structure	C5
				Energy consumption per unit of GDP	C6
	Socio-economic development system		Economic development subsystem	GDP growth rate	C7
				The proportion of added value of tertiary industry in GDP	C8
				Urbanization rate	C9
			Social development subsystem	Number of university students	C10
				Number of participants in basic medical insurance	C11
				Number of minimum living allowances for urban residents	C12

#### Low-carbon system indicators selection

According to the research content of this paper, the essence of low-carbon development is to improve resource utilization rate and reduce energy consumption rate. Therefore the model decomposes the low-carbon development system into energy environment subsystem and resource environment subsystem, and then selects its key indicators.

##### Resource environment subsystem

Forest cover, which is the ratio of forest area to total land area, is often used to measure the abundance of forest resources and ecological balance, and the increase of forest area helps to slow down the warming process. Air quality indicator good rate, calculated as the proportion of good air quality days to the total number of days, is closely related to the quality of the ecological environment. The garbage harmless treatment rate, which refers to the proportion of household garbage disposed of in a harmless manner, is a problem of various greenhouse gas emissions in the process of garbage disposal, and the higher the rate of harmless disposal, the lower the emission of methane and other greenhouse gases. Only by making every effort to increase the forest coverage area, improve the number of good air days and reduce the harmful incineration of garbage can we achieve the synergistic development of low-carbon and economic society.

##### Energy environment subsystem

The energy production structure can largely influence the energy consumption structure, carbon emission intensity and energy intensity. By reducing the proportion of coal in the energy structure and increasing the proportion of new and renewable energy, the goal of reducing carbon emissions per unit of GDP can be gradually accomplished. Energy consumption structure, using the composition of total energy consumption as a measure, directly affects carbon emission intensity, and the interaction term of renewable energy, energy investment, and energy investment and renewable energy also has a significant negative impact on CO_2_ emissions^17^. Energy consumption per unit GDP, which can measure energy use efficiency, has many influencing factors, and technological progress and scientific innovation promote economic growth and carbon intensity reduction^18^.

#### Socio-economic system indicators selection

##### Economic development subsystem

The GDP growth rate, which is an important measure of economic development level. In the process of economic growth, high-quality economic development is an important goal at this stage. The proportion of tertiary industry, one of the measurement elements of sustainable economic development. The urbanization rate, reflecting the degree of development of a city, will gradually increase with the improvement of social productivity and technological progress.

##### Social development subsystem

The number of college students in school and the talent environment are the soft power, which are the key elements to measure the level of social development. The number of basic medical insurance participants, employment and social security work is an important part of improving people's livelihood, and is an important pillar of social harmony and stability. The number of urban residents with minimum living security, an indicator to measure the level of economic and social development, the orderly development of low-carbon economy can indirectly increase the per capital life expectancy, improve people's livelihood and promote overall social progress.

### Synergy model construction

The theoretical core of synergy is used to analyze the ordered structure from the perspective of time, space and function through the internal synergy action of the system^19,20^. Based on the principle that "the synergy of many subsystems is governed by the same principle and had nothing to do with the characteristics of subsystems"^21^. So the two subsystems are constructed by low-carbon development system and socio-economic development system, and the inherent commonness and rules of the composite system formed by the interaction of the two subsystems according to the synergy theory are analyzed.

#### Indicator order degree analysis

In Table 1, each subsystem contains $n$ order parameters, and the system running is positively related to positive indicator. Instead of negative indicator, order parameter can be expressed as $h_{ji} = \left\{ {h_{j1} ,h_{j2} , \ldots h_{jn} } \right\}$. And then, every indicator and their system order degree model, as well as system synergy degree model are constructed.

Order degree represents the degree of components order of a system, which can be analogous to "entropy". According to the servitude principle of synergy theory, the order degree can be calculated by Eq. (1)^22^.

1 $$\sigma_{j} (h_{ji} ) = \left\{ {\begin{array}{*{20}ll} \frac{{h_{ji} - \min h_{ji} }}{{\max h_{ji} - \min h_{ji} }}, & \quad i \in \left( {1,k} \right) \\ \frac{{\max h_{ji} - h_{ji} }}{{\max h_{ji} - \min h_{ji} }}, & \quad i \in \left( {k + 1,n} \right) \\ \end{array} } \right.$$

where, $\sigma_{j} (h_{ji} )$ represents the order degree value of each indicator in the subsystem in different years, and $\sigma_{j} (h_{ji} ) \in \left[ {0,1} \right]$, whose value reflects the order degree of the system.

#### System order degree analysis

After the order degree value of each indicator is got by Eq. (1), the order degree measurement indicators of subsystems can be integrated by Eq. (2) from the order degree value of each indicator in different time periods through geometric average.

2 $$\mu_{j} (h_{j} ) = \sqrt[n]{{\prod\limits_{i = 1}^{n} {\mu_{j} (h_{ji} )} }}$$

#### Analysis of system coordination

The system coordination degree itself can be got by geometric average method. In order to measure the stability degree of the system in different time periods, the time dimension is innovative incorporated into the model construction by Eq. (3).

3 $$cor\left( {S_{1} ,S_{2} } \right) = \varpi \sqrt[n]{{\prod\limits_{j = 1}^{n} {\mu_{j}^{1} (h_{j} ) - \mu_{j}^{0} (h_{j} )} }}$$

where, $\varpi = \frac{{\min \left[ {\mu_{j}^{1} (h_{j} ) - \mu_{j}^{0} (h_{j} )} \right]}}{{\left| {\min \left[ {\mu_{j}^{1} (h_{j} ) - \mu_{j}^{0} (h_{j} )} \right]} \right|}}$ represents the action direction of subsystem on the synergy degree of composite system, and the value is -1 or 1. The synergy degree of composite system is represented via $cor\left( {S_{1} ,S_{2} } \right)$. Furthermore, its value range is $\left[ { - 1,1} \right]$. Its value is positively correlated with the degree of synergy. The synergy level corresponding to different values is divided into five equal sections according to the interval (shown in Table 2). The division of synergy level mainly refers to the research results of domestic scholars^23–25^.

**Table 2:** Synergy level division table of composite system.

Synergy degree of interval	Synergy level
[− 1, − 0.666]	Highly non-synergistic
[− 0.666, − 0.333]	Medium non-synergistic
[− 0.333, 0]	Mildly non-synergistic
[0, 0.333]	Mildly synergy
[0.333, 0.666]	Medium synergy
[0.666, 1]	Highly synergy

### R/S model analysis

The rescaled range analysis model is referred by R/S model. It is based on the study of different indicators in each subsystem of a nonlinear time series. And then, the Hurst exponent is got by using the concepts of standard deviation, range and logarithmic distribution^26^. The indicator is used to measure the persistence or anti-persistence of the time series in the studied system and to make a scientific prediction of their future development. Time series are used to the sequence formed by the numerical values of a certain statistical indicator at different times in chronological order^27^, which has been widely used in the study of quantitative prediction system. At present, rescaled range analysis model (R/S model), spectrum analysis model, periodic graph regression model and correlation analysis model are mostly used to analyze the fractal characteristics of time series^28–31^. However, the R/S model is of high accuracy to analyze the fractal structure characteristic of time series. So the model is conducive to the understanding of the subsystem between low-carbon development and socio-economic development.

The main steps of R/S model are given as follow:

1. The research sequence is scientifically divided into $n$ sub-sequences with length $m$. Where, $X_{j}$ represents as any time series. While,$j = 1,2, \ldots ,n$, and the element in $X_{j}$ is $X_{i,j}$,$i = 1,2, \ldots ,m$.
  Each time series have a corresponding mean and standard deviation series, which are got by Eqs. (4) and (5).
  4 $$E_{j} = \frac{1}{m}\sum\limits_{i = 1}^{m} {X_{i,j} }$$
  5 $$S_{j} = \sqrt {\frac{{\sum\nolimits_{i = 1}^{m} {\left( {X_{i,j} - E_{j} } \right)^{2} } }}{m - 1}}$$
2. Within each subset $j$, the cumulative deviation of the previous $i$ points ($i = 1,2, \ldots ,m$) is got by Eq. (6).
  6 $$C_{k,j} = \sum\limits_{i = 1}^{k} {\left( {X_{i,j} - E_{j} } \right)} ,\;\;\;k = 1,2, \ldots ,m$$
3. The range of fluctuation $R_{j}$ of each subset is got by Eq. (7).
  7 $$R_{j} = \max C_{k,j} - \min C_{k,j}$$
4. The rescaling range $R/S$ of each subset and the rescaling range $\left( {R/S} \right)_{m}$ on the time span of $m$ are calculated by Eqs. (8) and (9).
  8 $$R/S = \frac{{R_{j} }}{{S_{j} }}$$
  9 $$\left( {R/S} \right)_{m} = \frac{1}{n}\sum\limits_{j = 1}^{n} {\frac{{R_{j} }}{{S_{j} }}}$$
5. The value of $m$ starts from 2, then the rescaling range sequence $\left\{ {\left( {R/S} \right)_{m} } \right\}$ of time series with different time spans can be got.
6. According to the definition of Hurst index $H$, OLS linear regression can be performed by SPSS23.0. Later, Hurst index is got by Eq. (10).
  10 $$\ln R/S = H\ln c + H\ln m$$
7. In Hurst index, $H \in \left\{ {0,1} \right\}$: when, $H > 0.5$, all or part of the data met the positive correlation or long memory, which means that the future trend of time series is consistent with the past. The closer the value is to 1, the longer the long memory will be. When, $H = 0.5$, it means that each data in the sequence is independent and unrelated, and the development trend of the preceding sequence has nothing to do with the change trend of the following sequence. When, $H < 0.5$, all or part of the data meet negative correlation or anti-memory, which means that the overall trend in the future will be contrary to the past. The closer the value is to 0, the stronger the anti-memory will be^32^.
8. The variation trend of the dispersion degree of observed values will be obtained by the coefficient of variation.

## Results

### Results of synergy degree model

According to Eqs. (1), (2) and (3) above, the change of synergy degree among low-carbon development system, socio-economic development system and their composite system are got in Anhui Province from 2011 to 2020 (shown in Table 3).

**Table 3:** Synergy degree in Anhui Province from 2011 to 2020.

–	Synergy degree of low-carbon development system	Synergy degree of socio-economic development system	Synergy degree of composite system
2011	–	–	–
2012	0.030	0.041	0.035
2013	0.059	0.056	0.058
2014	0.086	0.075	0.081
2015	0.097	0.094	0.095
2016	0.106	0.109	0.107
2017	0.104	0.129	0.116
2018	0.110	0.157	0.132
2019	0.117	0.187	0.148
2020	0.125	0.218	0.165

In Table 3, based on the single system perspective, the low-carbon development system has been at a mild synergy level during 2011–2020, from 0.030 in 2012 to 0.125 in 2020. The synergy level of low-carbon development in Anhui Province has been increasing, although there is a small decline in the synergy level in 2016–2017. The slight fluctuation is the result of the synergy of both positive and negative effects brought by the implementation of relevant measures in the process of energy conservation and emission reduction. The synergy level of socio-economic development system is higher than low-carbon development, from 0.041 in 2012 to 0.218 in 2020, and the social development has been in a steady increase. Among them, the development was relatively flat from 2011–2016, but from 2017 to the present, the socio-economic development is in a rapid upward stage.

Based on the composite system perspective, during 2011–2016, the development trend of low carbon development system and socio-economic development system tends to be consistent and the synergy degree is high, and the two systems are at a mild level; from 2016 onwards, the marginal changes in the synergy degree of each system are more positive, and the obvious degree of change is ranked as follows: socio-economic development system, composite system, and low-carbon development system. By 2020, the synergy degree of the above three systems are 0.218, 0.165 and 0.125, respectively. Combined with the marginal changes of synergy degree, it can be seen that the composite system is at the level of light synergy and will soon enter the level of medium synergy, and the system self-organization tends to be solid.

### Results of R/S model

#### Variation coefficient

**Figure.2:**
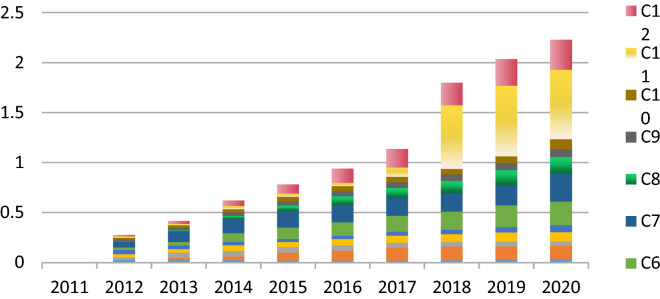
Variation trend chart in Anhui Province from 2011 to 2020.

The coefficient of variation is not affected by data dimension and is widely used to analyze the dispersion degree and average level of a variable in the system. In this study, the variation coefficients of 12 indicators between low-carbon development system and socio-economic development system are measured in Fig. 2. Among them, the variation of single indicator changes the most for the number of basic medical insurance participants in the evaluation system of low-carbon development in Anhui Province from 2011 to 2020. Medical insurance is the inevitable result of social progress and production development, as well as the external manifestation of labor productivity improvement and production development. The improvement of marginal change means that great efforts have been made on the construction of new social relations and social development accordingly. At the same time, the variation coefficients among three indicators, namely, the minimum subsistence allowance number of urban and rural residents, GDP growth rate and energy consumption per unit GDP, have similar trends. The changes of these three indicators indicate that more attention has been paid to social livelihood to effectively improve the quality of socio-economic growth and positively perfect the efficiency of resource utilization from 2011 to 2020 in Anhui Province. Otherwise, practical efforts have been made on the coordination between socio-economic development and low-carbon development. Furthermore, the coefficient of variation trend is not obvious for the forest coverage rate, it has been carrying out land greening actions, but the results can’t be completed overnight. After 30 years of hard work, it has reached the internationally recognized standard for evaluating good regional ecological conditions in 2020. From the trend of variation coefficient, the changes of the 12 indicators are in a steady increase, which means that it has been in the process of promoting the improvement of benefits in related fields.

#### R/S estimation results analysis

**Figure 3:**
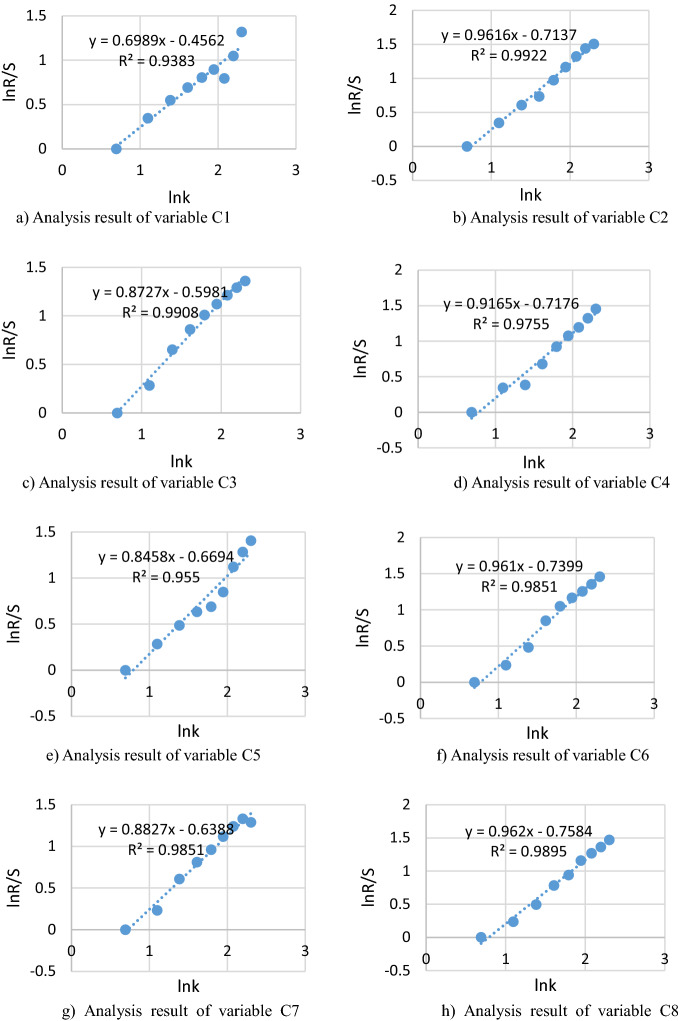

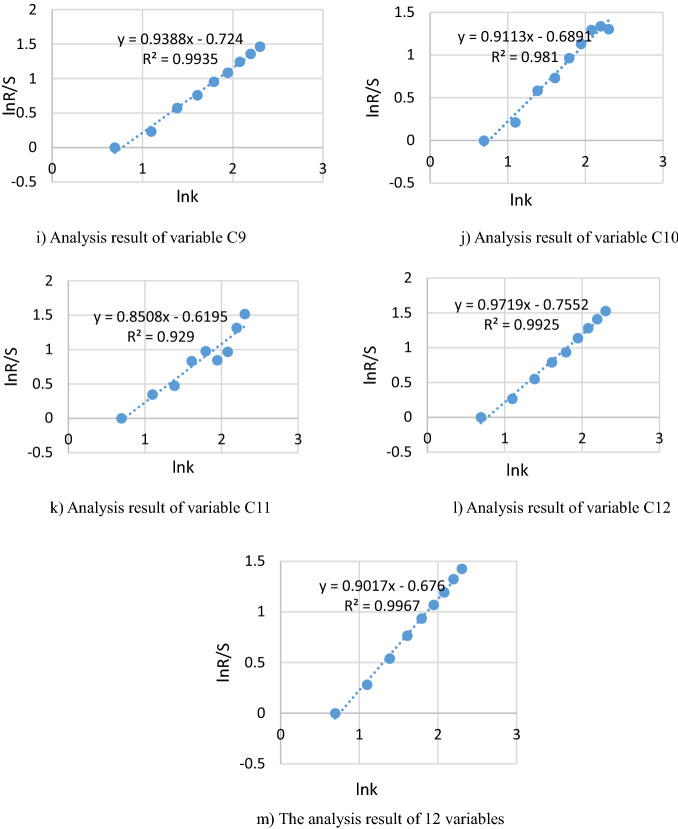
Results analysis in Anhui Province from 2011 to 2020.

According to the above calculation (Eqs. 4–10), combined with the variation characteristics of relevant order parameters in Anhui Province, the numerical values of 12 order parameters from 2011 to 2020 are analyzed by the corresponding $\ln (k)$ and $\ln (R/S)$, and the regression equation is fitted with the least square method by SPSS23.0 in the double logarithmic coordinate. Correlation curves and significance value $R^{2}$ are got in Fig. 3.

In R/S model, it has certain statistical significance to use $\ln (k)$ and $\ln (R/S)$ in 10 years to fit the line of low-carbon development system. Among them, the correlation coefficients between each order parameter and the composite system are $R^{2} > 0.9$, and the statistical analysis is $P < 0.5$, indicating that the regression effect is significant. Meanwhile, combined with the Hurst index and the basic principle of R/S model in Fig. 3, it can be known that H values of both single indicator and composite system meet the test criterion ($0.5 < H < 1$), which means that the time series studied in this study has long-term significant correlation, and the change of composite system between low-carbon development and socio-economic development in the future will inherit the development trend of the past, namely, the mild synergy development level will be continued. According to the trend analysis of synergy level of the composite system from 2011 to 2020, the synergy level shows an increasing trend year by year, which can predict that each order parameter in the system and the composite system will develop towards the medium synergy level in the future.

### Analysis of results

In the context of the current development situation of Anhui Province during 2011–2020, it is easy to find that the economy of Anhui Province maintains the momentum of continuous rapid and healthy development, the GDP growth rate continues to climb under the current economic policy, and the stability of economic growth is increasing. At the same time, the problem of urban development vulnerability has intensified, the economic development is overly dependent on energy consumption, the greenhouse gas emissions have increased significantly, and the climate change situation is becoming more and more severe. The situation of climate change is becoming increasingly serious. The contradiction between economic development and resources and environment is becoming increasingly prominent in China, and the low-carbon development system, socio-economic development system and the composite system have been at a mildly synergistic level, and great efforts are needed to enhance their synergy as soon as possible.

With the acceleration of the global climate governance process, the initiative is taken to announce that it would implement more stringent energy conservation and emission reduction policies, and it is announced at the Copenhagen Climate Conference that carbon dioxide emissions per unit GDP would be reduced by 40% in 2020 compared 45% in 2005^33–35^. Meanwhile, low-carbon development is gradually permeating all aspects between people's production and living activities. During the 12th Five-Year Plan Period (2011–2015), the integration process in Anhui Province and the Yangtze River Delta economic circle is significantly accelerated, the linkage of regional development is enhanced, and the coordination among eastern, central and western regions alleviates the environmental burden, which is the main reason why the coordination degree between low-carbon development and socio-economic development in Anhui Province is in the mild coordination stage.

After entering the period of the 13th Five-Year Plan (2016–2020), the socio-economic development in Anhui Province shows a trend of "faster speed, better structure, improved people's livelihood and enhanced vitality", laying a solid foundation for the good start of the 14th Five-year Plan^36^. Under the boost of the new normal of economic development, Anhui Province insists on improving the "green content" of ecological environment and "gold content" of economic development, firmly maintains the strategic determination to strengthen the construction of ecological civilization, promotes ecological governance and green industry in parallel, and promotes high-quality development with green development^37^. Therefore, the marginal synergy degree between low-carbon development system and socio-economic development system increases at different rates. Although the synergy level is still at the level of mild synergy development, but according to the principle of R/S model, the synergy level is changing to the level of medium.

## Discussions

This study combines the construction of synergy model and R/S analysis principle to verify the synergistic relationship between low-carbon development and socio-economic development, which has important value and inspiration for Anhui Province and even China to promote the synergy of pollution reduction and carbon efficiency, accelerate the construction of economic governance system and green low-carbon circular development economic system.Combined with the research results of this paper and the focus of academic circle on low-carbon economy, the following discussion points were formed.

### Promoting the coordinated efforts between "effective market" and "capable government" is necessary

From the perspective of win–win development, the coordinated efforts between "effective market" and "capable government" should be fully promoted, and the path of win–win cooperation between low-carbon development and socio-economic development should be unswervingly pursued^38^. Otherwise, low-carbon development should be based on the main contradiction in current society, such as committing the low-carbon transformation on the supply side, guiding low-carbon consumption to become a conscious behavior, and illuminating the road of improving the economy with the light of ecological civilization.

### Perfecting the fundamental transformation of resource utilization mode is unavoidable

From the perspective of resource flow, resources should be vigorously economized and utilized intensively to promote the fundamental transformation of resource utilization mode. Otherwise, the whole process of conservation management should be perfected, such as reducing the intensity of water and land consumption, strengthening the prevention and control of environment pollution, and improving its control in key industries and regions. Furthermore, the basic theory of environmental and resource adjudication should be strengthened. And then, a society in which resources and environment develop in harmony can be realized.

### Improving the transformation of energy structure with the advanced science and technology is inevitable

From the perspective of the energy flow, a reasonable energy structure should be established, such as improving the effect of energy conservation and consumption reduction, and promoting the energy transformation with the advanced science and technology as the support. Otherwise, the three major areas among effective energy development, clean energy and energy utilization should be focused, such as breaking through the key technological bottlenecks, supporting the green technology innovation, developing the low-carbon energy and applying more carbon-free energy. And then, a modern governance system that is low-carbon and mutually complementary can be built to enhance the independence and security of our energy.

## Summary and conclusions

In this study, a relatively recognized indicator system is constructed to analyze the situation of synergy between low-carbon development and socio-economic development in Anhui Province with a more targeted way. Then, the complicated conditions of low carbon development in Anhui Province are analyzed to help the realization of sustainable development. Furthermore, the synergy degree model and R/S model are integrated to achieve scientific analysis and research on the synergistic social, economic and environmental development in Anhui Province. In conclusion, the research results show that carbon emissions in Anhui Province have achieved significant results, and the growth rate of emissions has gradually decreased, making a great contribution to promoting sustainable development between low-carbon development and socio-economic development. In addition, some recommendations should be adopted as follows.

First, the science and technology policies should be firmly implemented to effectively support the socio-economic progress in Anhui Province. It means that not only the structural reform of production and supply side should be optimized, but also the green transformation of consumption mode should be accelerated.

Second, on the basis of the continuous improvement of ecological and environmental quality during the 13th Five-Year Plan period, environmental governance should be emphasized to free up the ecological and environmental capacity. And then, the transformation of low-carbon development from quantitative to qualitative change can be accelerated.

Finally, it is the right thing to learn from other countries’ achievements with the aim of the synergy between low-carbon development and socio-economic development. And then, the driving force and potential for sustainable development can be tapped and released continuously, the relationships among energy, environment and social benefits can be promoted, and the improvement of people’s livelihood can be achieved ultimately.

## Data Availability

All data generated or analyzed during this study are included in this article.
